# Malnutrition leads to the progression of coronary artery calcification in hemodialysis patients

**DOI:** 10.1371/journal.pone.0280383

**Published:** 2023-01-13

**Authors:** Hiroki Okabe, Yoshitaka Muraoka, Yutaro Naka, Koshi Setoyama, Konosuke Inoue, Toshiya Miura, Akiyoshi Shimizu, Reo Anai, Tetsu Miyamoto, Yuki Tsuda, Masaru Araki, Shinjo Sonoda, Masaharu Kataoka

**Affiliations:** 1 The Second Department of Internal Medicine, University of Occupational and Environmental Health, Kitakyushu, Japan; 2 Division of cardiology, Japan Labor Health and Welfare Organization Kyushu Rosai Hospital, Kitakyushu, Japan; 3 Department of Cardiovascular Medicine, Saga University, Saga, Japan; BSMMU: Bangabandhu Sheikh Mujib Medical University, BANGLADESH

## Abstract

**Background:**

Malnutrition is considered a risk factor for cardiovascular disease in patients with chronic kidney disease. However, no in vivo studies have reported on using optical coherence tomography to evaluate the effect of nutritional status on coronary atherosclerosis in hemodialysis patients. We aimed to conduct a detailed analysis of the effect of nutritional status on the coronary arteries in hemodialysis patients.

**Methods:**

Among 64 hemodialysis patients who underwent percutaneous coronary interventions, 41 that underwent optical coherence tomography imaging were included in this study. And, among them, 24 patients that could also be evaluated using OCT also at the 6-month follow-up were included in this study. The patients were divided into two groups based on nutritional evaluation using the geriatric nutritional risk index. Culprit and non-culprit lesions were evaluated at baseline and after 6 months.

**Results:**

In the culprit lesions at baseline, the length of the lipid plaque was significantly smaller in the malnutrition group. In contrast, the thickness and length of the calcified plaque and the angle of the calcified nodule were significantly larger (each p < 0.01). In the non-culprit lesions, the 6-month change in the angle of the calcified plaque was significantly greater in the malnutrition group (p = 0.02). The significant factors that affected the change in the angle of calcification were "malnutrition at geriatric nutritional risk index" [odds ratio, 8.17; 95% confidence interval, 1.79 to 37.33; p < 0.01] and "serum phosphorus level" (odds ratio, 3.73; 95% confidence interval, 1.42 to 9.81; p < 0.01).

**Conclusions:**

Appropriate management of nutritional status is crucial for suppressing the progression of coronary artery disease in hemodialysis patients.

## Introduction

Patients on hemodialysis (HD) are at a high risk for coronary artery disease (CAD), with most deaths originating due to cardiac causes [1]. These patients often undergo percutaneous coronary intervention (PCI); however, their prognosis remains poor [2]. Several previous studies have shown that HD patients have severe atherosclerosis, with a mortality risk up to 20 times higher than that in the age- and sex-matched general population [3].

Previously, traditional factors (e.g., older age, male sex, diabetes, and smoking) were considered to be the main risk factors for CAD in HD patients. Recently, non-traditional factors such as malnutrition, inflammation, and mineral and bone metabolism have also been studied [4]. With the growing number of long-term and older HD patients, the number of cases with malnutrition and wasting conditions has increased. In particular, for HD patients, control of malnutrition, frailty, and abnormalities in mineral and bone metabolism, such as serum phosphorus levels, are considered important because they affect the prognosis and vascular calcification [5,6]. Patients undergoing HD with malnutrition have poor all-cause and cardiovascular survival. Several reports have used computed tomography calcification scores to describe the effects of nutritional status on the prognosis and vascular calcification [7,8]. However, to the best of our knowledge, no previous study has evaluated the effect of nutritional status on coronary atherosclerosis in vivo.

Thus, in this study, we used optical coherence tomography (OCT) to assess plaque changes over time and evaluated the effect of nutritional status on coronary atherosclerosis in HD patients.

## Patients and methods

### Study design and patients

A prospective observational study was designed to observe culprit lesions and non-culprit lesions in HD patients who underwent OCT-guided percutaneous coronary intervention (PCI). OCT evaluation of the lesions was undertaken at baseline and after 6 months. The baseline was defined as the date of PCI, and the baseline OCT evaluation was performed before the PCI. We enrolled patients who had completed the follow-up examinations up to 6 months after the baseline measurements. This study included HD patients who received catheters between October 2017 and March 2021. The number of patients registered was as per the calculated sample size required for statistical purposes. The required sample size for statistical purposes was a total of 44 cases, 22 cases in each group. Therefore, the study first prospectively enrolled 45 cases of culprit lesions and 72 cases of non-culprit lesions. Data for the included patients were collected between October 2017 and September 2021 and then used to perform the analysis. No individual patient could be identified from the data collected. The study protocol was approved by the institutional review board of University of Occupational and Environmental Health (Kitakyushu, Japan). This research was conducted in accordance with the 1964 Declaration of Helsinki and amendments and as per the Ethical guidelines for life sciences and medical research involving human subjects presented by the Ministry of Health, Labour and Welfare. Therefore, informed consent was obtained from all patients.

**Fig 1:**
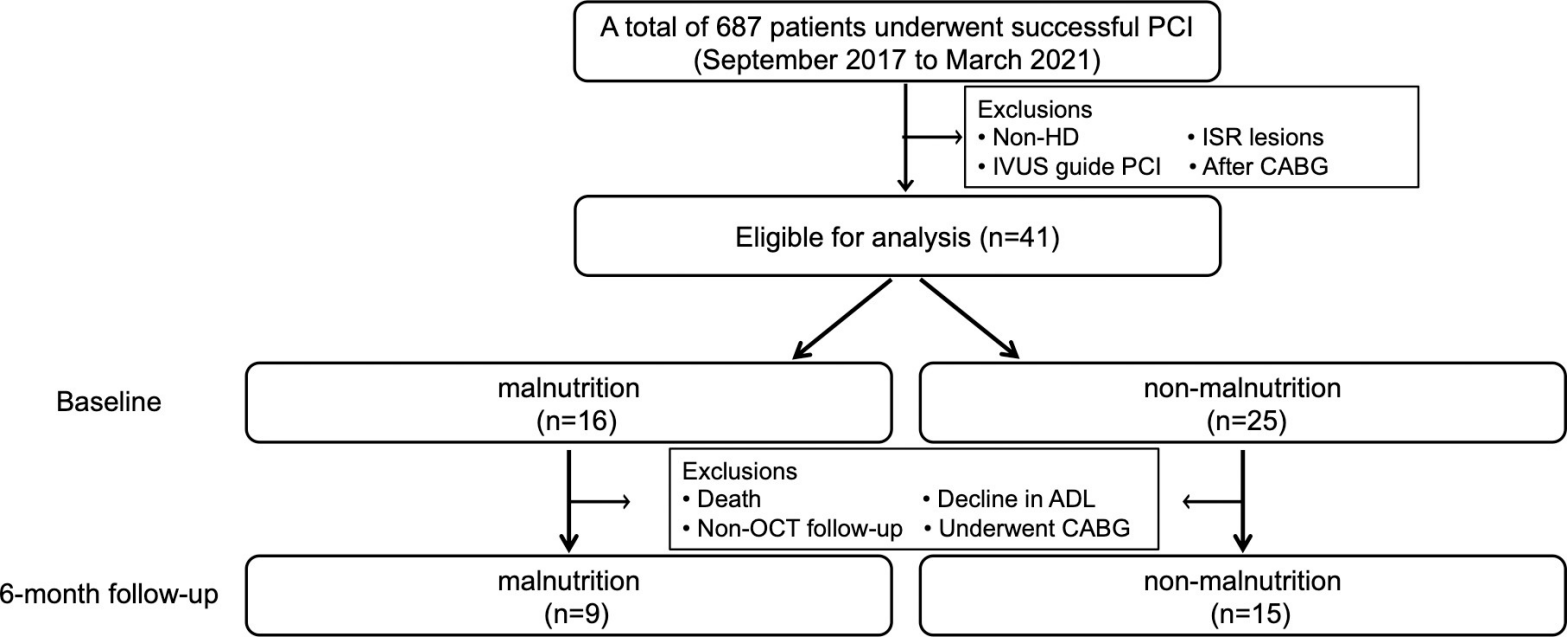
Description of non-culprit lesion.

Follow-up coronary angiography and OCT were performed after 6 months of standard medical therapy, including statin treatment and HD management with or without symptoms, according to the discretion of the attending physician. Segments 10 mm anterior and posterior to the minimal lumen area location, covering a total length of 20 mm, were included in the analysis. Non-culprit lesions were defined as 20-mm segments that were > 3 mm away from the ostium of each coronary artery and showed > 50% stenosis on quantitative coronary angiography. To eliminate the influence of stent edges, the target non-culprit lesions had to be at least 10 mm from the stented area if they were in the same coronary artery vessel (Fig 1).

Non-culprit lesions were defined as 20-mm segments that were located > 3 mm away from the ostium of each coronary artery and showed > 50% stenosis on quantitative coronary angiography. To eliminate the influence of stent edges, the segments that were located at least 10 mm away from the stent edges were adopted as non-culprit lesions, in cases in which stents existed in the same coronary artery vessels.

Dyslipidemia was controlled to ensure a low-density lipoprotein cholesterol level of 100 mg/dL and a high-density lipoprotein cholesterol level of 40 mg/dL. Unless contraindicated, statins were prescribed to patients who could tolerate them to manage their lipid profiles to the target control levels. Diabetes mellitus was defined as a glycated hemoglobin level > 7.0%. Hypertension was defined as a systolic blood pressure > 130 mmHg and diastolic blood pressure > 80 mmHg.

### Nutritional status

Patients were classified into two groups according to their geriatric nutritional risk index (GNRI) values. This index was calculated using the serum albumin level and body weight with the following equation:

GNRI=[1.489×albumin(g/dL)]+[41.7×(bodyweight/idealbodyweight)] (1)

When the patient’s body weight exceeded the ideal body weight, it was set to 1. In this study, the ideal body weight was defined as the value calculated from the height and a body mass index (BMI) of 22 because of its validity [9], instead of the value calculated with the Lorentz formula used in the original GNRI equation. Yamada et al. reported that 91.2 was the standard for nutritional assessment in patients undergoing HD [5]. Thus, here, patients with GNRI values < 91.2 were included in the malnutrition group, while those with GNRI values ≥ 91.2 were included in the non-malnutrition group. Body measurements and bioelectrical impedance analysis were used to measure musculoskeletal muscle mass and body fat percentage as supplementary items for nutritional assessment.

### OCT imaging

OCT imaging was performed using a frequency-domain OCT system (C8 System, Dragonfly Imaging Catheter, and ILUMIEN OPTIS; St. Jude Medical, St. Paul, MN, USA) and an optical frequency-domain imaging system (LUNAWAVETM and a FastviewTM system; Terumo Corporation, Tokyo, Japan). After intracoronary administration of 1–2 mg isosorbide nitrate, a 2.7-Fr (Dragonfly OPTIS: St. Jude Medical, USA) or 2.6-Fr catheter (Fastview: Terumo Corporation, Japan) was pulled back from the distal site of the culprit lesion for OCT imaging in the frequency domain. The pullback was triggered automatically using the intracoronary contrast injection (3–4 mL/s, 12–14 mL total), with a maximum motorized pullback speed of 25 mm/s (Dragonfly) or 40 mm/s (Fastview), a frame rate of 100/s (Dragonfly) or 160/s (Fastview), and a maximum scan length of 75 mm (Dragonfly) or 150 mm (Fastview).

### Measurements of parameters

**Fig 2:**
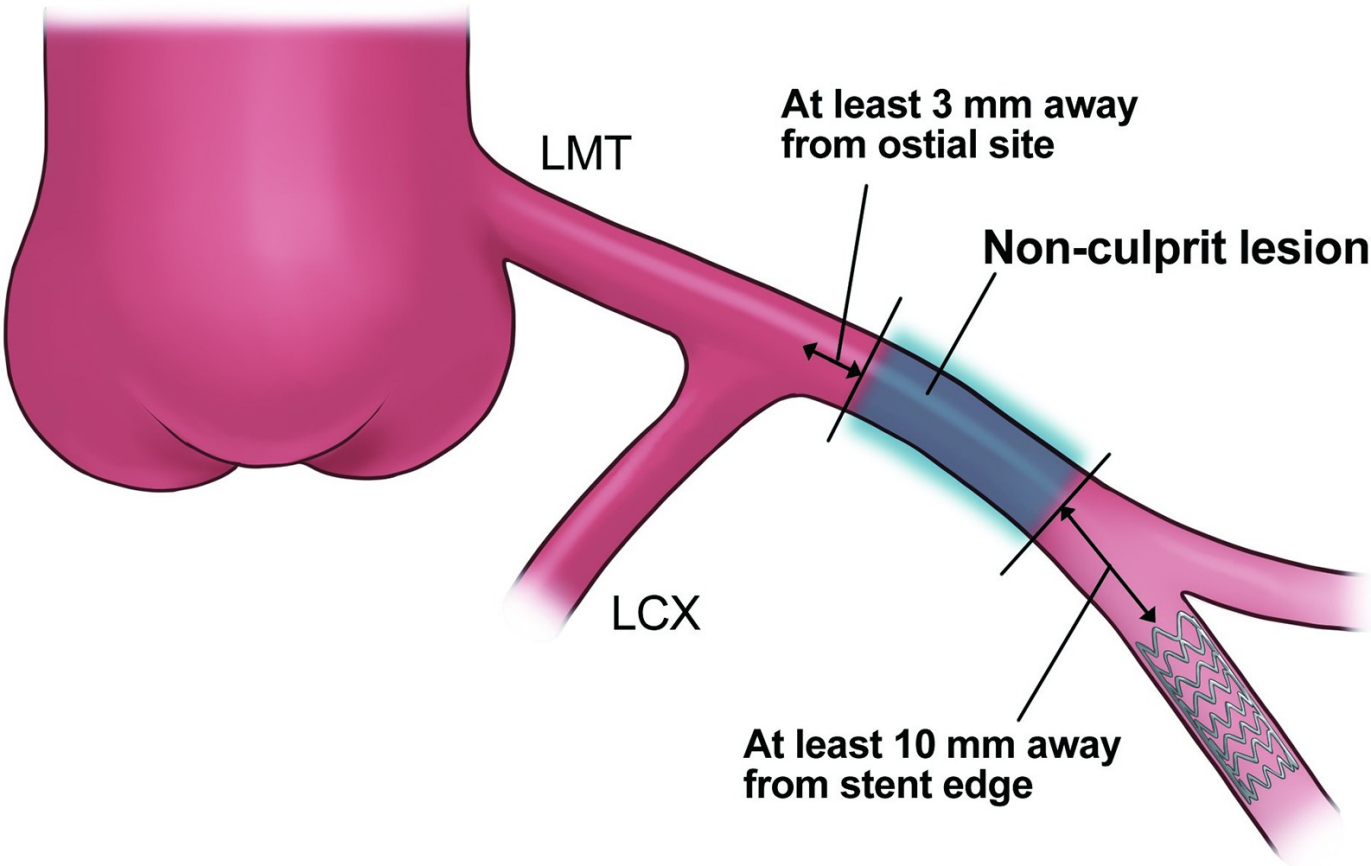
Description of quantitative assessment by OCT.

All OCT images were analyzed by two experienced investigators using a dedicated software (St. Jude Medical Inc. or Terumo) and previously validated criteria for OCT plaque characteristics [10]. In cases involving disagreement, a consensus was reached after consulting a third researcher. Six months later, follow-up OCT examinations were repeated on the same coronary segments imaged at the baseline examination, regardless of the presence of clinical symptoms. Image correspondence was confirmed on the basis of the distances from nearby side branches. A calcified plaque was defined as a poorly signaled or heterogeneous area with well-defined boundaries, and a calcified nodule was defined by the accumulation of nodular calcification (small calcium deposits) with disruption of the fibrous cap on the calcification plate. Quantitative analysis of the calcification was performed at 1-mm intervals throughout the lesion, and the three parameters of maximum angle, maximum thickness, and length were used to evaluate each calcium deposit in the target lesion. The thickness of the calcification was analyzed in the slice with the largest angle. The calcification length was calculated by multiplying the total number of slices containing calcification by the frame interval (Fig 2). Finally, in cases involving multiple calcified deposits, the deposit with the largest angle of calcification was considered to indicate the calcification of the target lesion, and the maximum angle, thickness, and length of the calcification were representative of each lesion [11]. Calcified nodules were defined as the maximum angle from the lumen present in the lesion, which was representative of each lesion. Lipid plaques were defined as those with diffuse boundaries in low-signal areas, and the maximum angle and length of the lipid arc were measured. Thin-cap fibroatheroma was defined as a lipid-rich plaque with a fibrous cap thickness of less than 70 μm [10]. Plaque rupture, thrombus formation, macrophage accumulation, and microchannels were identified using previously described methods [10]. Additionally, after stent implantation, OCT evaluation of culprit lesions at 6 months and qualitative evaluation of in-stent neointima were performed on the basis of previous reports [12,13].

The maximum angular portion of each plaque was measured. The asterisk indicates a calcified nodule. OCT, optical coherence tomography.

### Statistical analysis

All data are expressed as median (interquartile range) or mean ± standard deviation. Differences in continuous variables were compared using a paired t-test; differences between groups were assessed using the chi-square test for categorical variables and the unpaired Wilcoxon rank-sum test for continuous variables. A comparison of plaque quantification parameters between the two groups was performed using the Wilcoxon rank-sum test at baseline and at 6 months. Changes in the plaque were also compared using the Wilcoxon rank-sum test. In this study, the values above the median were defined as having plaque progression at each of the plaque changes. Univariate nominal logistic regression analysis was performed to identify the predictors of plaque progression. The association between nutritional status and coronary plaque change after adjustment for conventional risk factors and medications was summarized as an odds ratio estimated using multivariate logistic regression analysis. Statistical significance was set at p < 0.05. Statistical analyses were performed using the JMP Pro version 15.2.0 (SAS, Cary, NC, USA).

## Results

### Study population

**Fig 3:**
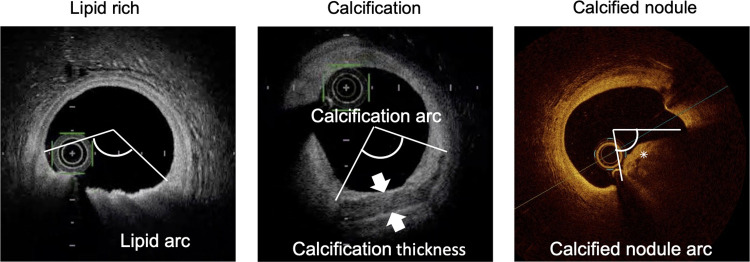
Flow chart of the study design. ADL, activities of daily living; CABG, coronary artery bypass grafting; HD, hemodialysis; ISR, in-stent restenosis; IVUS, intravascular ultrasound; OCT, optical coherence tomography; PCI, percutaneous coronary intervention.

Fig 3 shows the breakdown of the patient population included in this study: 687 consecutive patients underwent PCI between October 2017 and March 2021 (Fig 3). Among these, 64 patients (89 lesions) also underwent maintenance HD, of which, 41 patients (45 lesions) underwent OCT-guided PCI. Of the 45 lesions, four with inadequate OCT imaging data were excluded and the remaining patients with 41 culprit lesions initially underwent OCT analysis before PCI. Of these, two patients whose non-culprit lesions could not be identified were excluded. Additionally, we excluded patients who underwent follow-up examinations by other methods (five patients), patients who could not be followed up due to death (three patients) or decline in activities of daily living (four patients), and patients who required a coronary artery bypass grafting procedure (three patients). We used serial OCT images of 25 culprit lesions and 47 non-culprit lesions (24 patients) obtained using the same type of OCT catheter at both baseline and follow-up for analysis. Of these, four culprit lesions and eight non-culprit lesions that could not be analyzed due to insufficient OCT imaging data were excluded. Finally, 25 culprit lesions and 39 non-culprit lesions in 24 patients could also be evaluated using OCT at the 6-month follow-up. Culprit lesions included 11 lesions in the malnutrition group and 14 lesions in the non-malnutrition group, while non-culprit lesions included 17 lesions in the malnutrition group and 22 lesions in the non-malnutrition group.

### Baseline clinical characteristics

The baseline clinical characteristics are shown in Table 1. Based on the factors of the GNRI formula, the BMI and serum albumin levels were significantly lower in the malnutrition group (each p < 0.01). furthermore, the left ventricular ejection fraction, arm circumference, arm muscle circumference, arm muscle area, and calf circumference were lower in the malnutrition group. The other parameters showed no significant differences between the groups.

**Table 1:** Baseline clinical characteristics of study participants.

Variables	Overall, n = 41	Malnutrition group, n = 16	Non-malnutrition group, n = 25	p value
Age (years)	71 (66−76)	74 (67−78)	69 (66−75)	0.19
Sex, male, n (%)	33 (80)	12 (75)	21 (84)	0.48
Body mass index	21.5 (19.1−24.1)	19.1 (17.0−21.1)	23.0 (21.1−25.4)	<0.01
HD periods (month)	44 (3−96)	11 (2−176)	44 (4−84)	0.90
GNRI	95.0 ± 14.7	82 ± 8	103 ± 12	<0.01
SMI (kg/m^2^)	6.4 (6.0−7.3)	6.1 (6.0−6.5)	6.4 (5.9−7.4)	0.20
Body fat percentage (%)	19.4 (15.9−23.7)	18.3 (14.4−20.6)	21.3 (15.9−31.8)	0.06
Muscle mass (kg)	42.0 (38.5−44.0)	40.9 (39.3−42.5)	42.4 (37.4−44.4)	0.37
AC (cm)	23.0 (22.2−25.0)	22.5 (19.8−24.4)	24.0 (22.6−25.7)	0.02
AMC (cm)	21.2 (20.1−22.6)	20.7 (18.3−21.9)	21.6 (20.5−23.0)	0.06
AMA (cm^2^)	35.4 (31.8−40.3)	32.6 (23.9−38.2)	36.8 (33.4−41.2)	0.04
TSF (mm)	8.0 (6.0−8.0)	6.0 (6.0−8.0)	8.0 (6.0−8.0)	0.08
CC (cm)	30.2 (28.2−32.8)	28.3 (25.7−31.5)	31.3 (29.7−33.5)	0.01
Hypertension, n (%)	36 (88)	13 (81)	23 (92)	0.30
Diabetes Mellitus, n (%)	24 (59)	8 (50)	16 (64)	0.37
Dyslipidemia, n (%)	16 (39)	7 (44)	9 (36)	0.62
Current smoker, n (%)	25 (61)	10 (63)	15 (60)	0.87
Peripheral Arterial disease, n (%)	13 (32)	7 (44)	6 (24)	0.18
Prior PCI, n (%)	13 (32)	4 (25)	9 (36)	0.46
History of cerebral infarction, n (%)	12 (29)	6 (38)	6 (24)	0.35
History of malignancy, n (%)	5 (12)	4 (25)	1 (4)	0.045
LVEF (%)	43.0 (35.0−51.0)	36.5 (30.0−42.0)	50.0 (37.5−55.0)	0.01
Aspirin, n (%)	41 (100)	16 (100)	25 (100)	-
P2Y12 receptor antagonist, n (%)	38 (93)	14 (88)	24 (96)	0.31
ACE-I/ARB, n (%)	23 (56)	9 (56)	14 (56)	0.99
β-blocker, n (%)	24 (59)	10 (63)	14 (56)	0.68
Statins, n (%)	37 (90)	13 (81)	24 (96)	0.12
Insulin, n (%)	12 (29)	4 (25)	8 (32)	0.45
White blood cell (×10^3^/μL)	6.9 (6.2−7.7)	6.6 (5.1−9.4)	6.5 (4.8−8.8)	0.61
Hemoglobin (g/dL)	11.3 (10.9−11.7)	11.1 (9.5−12.4)	11.6 (10.7−12.8)	0.22
Serum albumin (g/dL)	3.7 (3.2−3.9)	3.1 (2.8−3.5)	3.8 (3.7−4.0)	<0.01
Serum Ca (mg/dl)	9.3 ± 0.7	9.5 ± 0.8	9.2 ± 0.6	0.26
Serum P (mg/dl)	4.5 ± 1.2	4.4 ± 1.4	4.6 ± 1.0	0.64
Ca×P	39.5 (25.8−50.9)	40.1 (31.1−55.2)	39.4 (35.4−49.3)	0.78
Intact PTH (pg/mL)	106.0 (62.5−198.0)	112.5 (60.2−200.2)	101.0 (62.5−199.5)	0.80
Serum creatinine (mg/dL)	5.9 (4.8−7.4)	5.4 (3.7−7.5)	6.0 (5.1−7.4)	0.29
High sensitive CRP (mg/dL)	0.2 (0.1−1.1)	0.8 (0.1−2.1)	0.2 (0.1−0.4)	0.11
Total cholesterol (mg/dL)	131.5 (116.5−164.0)	131.5 (120.0−156.0)	129.0 (114.5−169.0)	0.98
LDL-cholesterol (mg/dL)	68.0 (54.0−87.5)	65.0 (55.2−84.8)	68.0 (52.0−99.5)	0.81
Triglyceride (mg/dL)	94.0 (57.0−134.0)	87.5 (57.0−104.5)	94.0 (66.5−131.5)	0.51
HbA1c (%)	5.7 (5.3−6.5)	5.5 (4.9−6.3)	5.9 (5.4−6.8)	0.11
Target lesions				
Culprit lesion	41 lesions	17 lesions	24 lesions	
LAD, n (%)	27 (66)	13 (76)	14 (58)	0.19
LCX, n (%)	4 (10)	0 (0)	4 (17)	
RCA, n (%)	10 (24)	4 (24)	6 (25)	
Non-culprit lesion	72 lesions	31 lesions	41 lesions	
LAD, n (%)	23 (32)	9 (29)	14 (34)	0.90
LCX, n (%)	4 (38)	12 (39)	15 (37)	
RCA, n (%)	22 (31)	10 (32)	12 (29)	

Values are described as number (%), median (interquartile range), or mean ± standard deviation.

ARB, angiotensin II receptor blocker; ACE-I, angiotensin-converting enzyme inhibitor; AC, arm circumference; AMC, arm muscle circumference; AMA, arm muscle area; Ca, calcium; CC, calf circumference; CI, confidence interval; CRP, C-reactive protein; GNRI, geriatric nutritional risk index; HD, hemodialysis; HR, hazard ratio; LAD, left anterior descending artery; LCX, left circumflex; LDL, low density lipoprotein; LVEF, left ventricular ejection fraction; P, phosphorus; PCI, percutaneous coronary intervention; PTH, parathyroid hormone; RCA, right coronary artery; SMI, skeletal muscle index; TSF, triceps skinfold.

### Baseline OCT analysis

Table 2 shows the baseline OCT findings. Seventeen lesions in the malnutrition group and 24 in the non-malnutrition group were enrolled and evaluated at baseline. Almost all HD patients had calcified plaques. Erosion and calcified nodules were found in a higher percentage of patients in the malnutrition group. In the quantitative evaluation of plaques, lipid plaques were larger in the non-malnutrition group, while calcified plaques and calcified nodules were larger in the malnutrition group. The length of the lipid plaque was significantly longer in the non-malnutrition group than in the malnutrition group (p < 0.01). For calcified plaques, the thickness and length were significantly greater in the malnutrition group (each p < 0.01), and for calcified nodules, the angle was significantly larger in the malnutrition group (p < 0.01).

**Table 2:** Baseline pre-PCI OCT findings of the culprit lesions.

Variables	Malnutrition group, n = 17 lesions	Non-malnutrition group, n = 24 lesions	p value
MLA (mm^2^)	1.6 (1.3−2.0)	1.2 (0.8−2.3)	0.14
Plaque rupture, n (%)	3 (18)	3 (50)	0.65
Erosion, n (%)	10 (59)	5 (21)	0.01
Lipid rich plaque, n (%)	12 (71)	22 (92)	0.08
TCFA, n (%)	2 (12)	4 (17)	0.66
Macrophage infiltration, n (%)	15 (88)	22 (92)	0.72
Microchannel, n (%)	13 (76)	19 (79)	0.84
Cholesterol crystal, n (%)	7 (41)	14 (58)	0.28
Thrombus, n (%)	8 (47)	8 (33)	0.37
Calcification, n (%)	17 (100)	23 (96)	0.39
Calcified nodule, n (%)	11 (65)	4 (17)	<0.01
Healed plaque, n (%)	4 (24)	6 (25)	0.91
Lipid rich plaque			
maximum plaque angle (°)	109.4 (37.2−176.1)	162.5 (92.0−265.1)	0.19
lipid length (mm)	2.3 (0.7−4.0)	5.0 (2.5−7.9)	<0.01
Calcification			
arc (°)	252.8 (133.2−360.0)	170.4 (100.0−250.8)	0.08
thickness (mm)	1.2 (0.9−1.4)	0.8 (0.7−1.1)	<0.01
length (mm)	16.3 (7.8−18.0)	5.8 (3.3−8.1)	<0.01
Calcified nodule			
arc (°)	63.5 (0−98.35)	0 (0−0)	<0.01

Values are described as number (%) or median (interquartile range).

MLA, minimal lumen area; TCFA, thin-cap fibroatheroma; PCI, percutaneous coronary intervention; OCT, optical coherence tomography.

### Clinical characteristics at the 6-month follow-up

The results of the analysis are shown only for patients who were able to undergo OCT follow-up at 6 months. In the analyses of patient background factors, the malnutrition group showed a lower left ventricular ejection fraction, serum albumin level, and BMI. The two groups showed no significant differences in other parameters (Table 3).

**Table 3:** Baseline clinical characteristics of patients who were followed up for 6 months.

Variables	Overall, n = 24	Malnutrition group, n = 9	Non-malnutrition group, n = 15	p value
Age (years)	72 (66−76)	71 (58−76)	73 (68−77)	0.37
Sex, male, n (%)	21 (88)	8 (89)	13 (87)	0.87
Body mass index	21.1 (18.8−24.3)	18.9 (17.0−20.1)	23 (20.7−25.7)	<0.01
HD periods (month)	34 (2−90)	88 (2−300)	24 (3−63)	0.61
GNRI	96 ± 16	82 ± 8	104 ± 13	<0.01
SMI (Kg/m^2^)	6.2 (6.0−7.4)	6.1 (6.0−6.9)	6.4 (6.0−7.5)	0.34
Body fat percentage (%)	18.9 (15.2−22.9)	17.8 (12.0−21.0)	20.9 (15.9−30.3)	0.17
Muscle mass (Kg)	41.6 (37.7−43.8)	40.8 (38.2−42.8)	41.6 (36.7−44.0)	0.65
AC (cm)	24.1 (22.1−25.3)	22.5 (20.0−24.8)	24.5 (23.0−26.0)	0.08
AMC (cm)	21.5 (20.2−22.8)	21.5 (19.1−22.3)	21.5 (20.5−23.0)	0.44
AMA (cm^2^)	35.8 (30.1−40.6)	35.9 (26.1−39.4)	35.6 (33.4−41.7)	0.34
TSF (mm)	8.0 (6.0−8.0)	6.0 (5.0−8.0)	8.0 (6.0−12.0)	0.08
CC (cm)	31.2 (29.0−32.9)	29.5 (26.7−32.0)	31.8 (30.2−34.0)	0.09
Hypertension, n (%)	21 (88)	7 (78)	14 (93)	0.26
Diabetes Mellitus, n (%)	14 (58)	5 (56)	9 (60)	0.83
Dyslipidemia, n (%)	11 (46)	4 (44)	7 (47)	0.92
Current smoker, n (%)	13 (56)	6 (67)	7 (47)	0.43
Peripheral Arterial disease, n (%)	3 (13)	2 (22)	1 (7)	0.26
Prior PCI, n (%)	6 (25)	2 (22)	4 (27)	0.81
History of cerebral infarction, n (%)	9 (38)	5 (56)	4 (27)	0.16
History of malignancy, n (%)	2 (8)	2 (22)	0 (0)	0.06
LVEF (%)	50.0 (35.8−55.0)	38.0 (32.5−46.0)	50.0 (50.0−55.0)	<0.01
Aspirin, n (%)	24 (100)	9 (100)	15 (100)	-
P2Y12 receptor antagonist, n (%)	23 (96)	8 (89)	15 (100)	0.19
ACE-I/ARB, n (%)	23 (56)	9 (56)	14 (56)	0.99
β-blocker, n (%)	10 (42)	5 (56)	5 (33)	0.29
Statins, n (%)	22 (92)	8 (89)	14 (93)	0.70
White blood cell (×10^3^/μL)	6.4 (4.7−8.0)	6.4 (4.4−8.0)	6.5 (4.7−8.8)	0.86
Hemoglobin (g/dL)	11.4 (10.4−12.1)	11.3 (9.4−12.2)	11.5 (10.6−12.1)	0.37
Serum albumin (g/dL)	3.7 (3.5−4.0)	2.9 (2.8−3.7)	3.9 (3.7−4.1)	<0.01
Serum Ca (mg/dL)	9.2 (8.9−9.6)	9.3 (8.9−10.5)	9.1 (8.9−9.4)	0.32
Serum P (mg/dL)	4.2 (3.7−5.5)	5.4 (3.6−6.1)	4.2 (3.7−4.8)	0.26
Ca×P	38.5 (33.1−51.8)	50.2 (32.8−60.7)	38.4 (32.9−47.0)	0.19
Intact PTH (pg/mL)	112.5 (60.8−177.5)	119.0 (66.0−186.5)	101.0 (59.0−178.0)	0.91
Serum creatinine (mg/dL)	5.9 (5.0−7.5)	6.4 (4.5−7.8)	5.7 (5.0−7.3)	0.61
High sensitive CRP (mg/dL)	0.13 (0.06−0.76)	0.14 (0.05−2.65)	0.11 (0.07−0.26)	0.61
Total cholesterol (mg/dL)	136.0 (113.0−156.0)	135.0 (112.0−154.0)	138.5 (114.8−194.8)	0.55
LDL-cholesterol (mg/dL)	68.5 (55.3−87.8)	61.0 (57.5−82.0)	72.0 (52.0−114.0)	0.70
Triglyceride (mg/dL)	94.0 (57.0−132.8)	69.0 (51.5−127.5)	94.0 (59.0−134.0)	0.42
HbA1c (%)	5.8 (5.3−6.9)	5.6 (4.6−6.2)	6.0 (5.3−6.9)	0.13
Target lesions				
Culprit lesion	25 lesions	11 lesions	14 lesions	
LAD, n (%)	17 (68)	9 (82)	8 (57)	0.23
LCX, n (%)	3 (12)	0 (0)	3 (21)	
RCA, n (%)	5 (20)	2 (18)	3 (21)	
Non-Culprit lesion	39 lesions	17 lesions	22 lesions	
LAD, n (%)	12 (31)	3 (18)	9 (41)	0.29
LCX, n (%)	16 (41)	8 (47)	8 (36)	
RCA, n (%)	11 (28)	6 (35)	5 (23)	

Values are described as number (%), median (interquartile range), or mean ± standard deviation.

ARB, angiotensin II receptor blocker; ACE-I, angiotensin-converting enzyme inhibitor; AC, arm circumference; AMC, arm muscle circumference; AMA, arm muscle area; Ca, calcium; CC, calf circumference; CI, confidence interval; CRP, C-reactive protein; HD, hemodialysis; HR, hazard ratio; LAD, left anterior descending artery; LCX, left circumflex; LDL, low density lipoprotein; LVEF, left ventricular ejection fraction phosphorus; PCI, percutaneous coronary intervention; PTH, parathyroid hormone; RCA, right coronary artery; SMI, skeletal muscle index; TSF, triceps skinfold.

### OCT analyses at the 6-month follow-up

We evaluated the neointima in the culprit lesions after stenting. We compared 11 lesions of the malnutrition group with 14 lesions of the non-malnutrition group (Table 4). Among the neoatherosclerosis cases, only the presence of thrombus was significantly more common in the malnutrition group. Although the groups showed no significant differences, the frequency of calcification tended to be higher in the malnutrition group. We compared the non-culprit lesions between the two groups by considering 17 and 22 lesions in the malnutrition and non-malnutrition groups, respectively (Table 5). Plaque quantification was determined at baseline and at 6 months. At each time point, lipid plaques tended to be larger in the non-malnutrition group, whereas calcified plaques tended to be larger in the malnutrition group, but no significant difference was observed. Only the angle of calcified nodule at 6-months was significantly greater in the malnutrition group. Intergroup comparisons of the amount of plaque change showed significant differences in lipid plaque length [0 (-2.2−0) vs. 0 (-0.4−2.3), p = 0.03] and calcified plaque angle [14.4 (10.4−24.5) vs. 4.9 (-0.9−19.4), p < 0.01]. The median change in the calcified plaque angle was 10.7°; therefore, a change of > 10° after 6 months was defined as significant plaque progression. In the univariate nominal logistic analysis to examine the influencing factors, "malnutrition as assessed by GNRI" [odds ratio (OR): 8.17; 95% confidence interval (CI): 1.79 to 37.33; p < 0.01] and “serum phosphorus level” (OR: 3.73; 95% CI: 1.42 to 9.81; p < 0.01) were significantly related to each other. Next, we performed a multivariate nominal logistic analysis with other factors known to influence calcified plaques, including the two factors described above [14–17]. Both multivariate analysis of “malnutrition as assessed by GNRI” and “serum phosphorus level,” and multivariate analysis of other factors showed that these two factors were significantly related (Table 6). We performed the same analysis for "lipid plaque length." Since the median length was 0.7 mm, we performed a univariate nominal logistic analysis, defining the group with significant plaque progression as the group in which the difference in length between the 6-month and baseline values was greater than 0 mm. In the univariate analysis, malnutrition as assessed by GNRI, BMI, and arm circumference were found to be significant influencing factors. However, when the multivariate nominal logistic analysis was conducted, none of them showed significance (Table 7).

**Table 4:** OCT findings of culprit lesions at 6-months follow-up.

Variables	Malnutrition group, n = 11 lesions	Non-malnutrition group, n = 14 lesions	p value
Neointima			
Homogeneous, n (%)	9 (82)	10 (71)	0.55
Heterogeneous, n (%)	5 (45)	6 (43)	0.89
Layered, n (%)	1 (9)	2 (14)	0.69
Neoatherosclerosis			
Uncovered strut, n (%)	4 (36)	3 (21)	0.41
Plaque rupture, n (%)	2 (18)	0 (0)	0.09
Lipid-rich, n (%)	0 (0)	0 (0)	-
Macrophage, n (%)	3 (27)	3 (21)	0.73
Microvessel, n (%)	3 (27)	1 (7)	0.17
Thrombus, n (%)	3 (27)	0 (0)	0.04
Calcification, n (%)	5 (45)	2 (14)	0.08

Values are described as number (%).

OCT, optical coherence tomography.

**Table 5:** OCT findings of non-culprit lesions.

Variables	Baseline			6-months			Differences within 6-months		
	Malnutrition group, n = 17 lesions	Non-malnutrition group, n = 22 lesions	p value	Malnutrition group, n = 17 lesions	Non-malnutrition group, n = 22 lesions	p value	Malnutrition group, n = 17 lesions	Non-malnutrition group, n = 22 lesions	p value
MLA (mm^2^)	3.4 (2.2−6.2)	4.1 (2.4−5.5)	0.98	3.4 (2.3−7.9)	4.0 (2.3−5.5)	0.88	0 (-0.7−0.3)	-0.1 (-0.2−0.1)	0.73
Lipid rich plaque									
maximum plaque angle (°)	109.5 (0−147.9)	108.7 (0−166.8)	0.75	0 (0−152.7)	110 (0−153.8)	0.37	0 (-31.3−1.3)	0 (-31.8−22.4)	0.77
lipid length (mm)	3.1 (0−5.2)	3.2 (0−5.5)	0.97	0 (0−6.9)	3.6 (0−7.4)	0.17	0 (-2.2−0)	0 (-0.4−2.3)	0.03
Calcification									
arc (°)	110.0 (63.0−266.1)	94.2 (59.2−106.6)	0.12	121.5 (80.4−322.6)	99.7 (62.9−114.1)	0.06	14.4 (10.4−24.5)	4.9 (-0.9−19.4)	<0.01
thickness (mm)	1.04 (0.26−1.16)	0.73 (0.48−1.06)	0.26	1.17 (0.46−1.28)	0.91 (0.69−1.04)	0.12	0.12 (0.05−0.19)	0.09 (0.02−0.16)	0.45
length (mm)	7.1 (2.6−10.8)	3.2 (1.8−5.9)	0.11	8.1 (3.3−15.2)	5.0 (2.0−7.0)	0.09	0.5 (0.1−3.7)	0.3 (-0.1−1.1)	0.36
Calcified nodule									
arc (°)	0 (0−29.9)	0 (0−0)	0.10	36.8 (0−58.4)	0 (0−9.2)	0.01	5.6 (0−42.6)	0 (0−0)	0.05

Values are described as median (interquartile range).

MLA, minimum lesion area; OCT, optical coherence tomography.

**Table 6:** Predictors for progression of vascular calcification.

Variables	Univariable analysis			Multivariable analysis		
	OR	95% CI	p value	OR	95% CI	p value
Malnutrition of GNRI	8.17	1.79−37.33	<0.01	6.23	1.17−33.32	0.03
Age	1.00	0.92−1.08	0.99			
BMI	0.96	0.81−1.13	0.60			
HD periods	1.00	0.99−1.01	0.51			
Right coronary	1.52	0.36−6.37	0.57			
Left anterior descending artery	0.69	0.18−2.70	0.59			
Left circumflex artery	0.99	0.27−3.58	0.99			
Hypertension	1.95	0.37−10.2	0.43			
History of malignancy	-	-	1.00			
Current smoker	0.79	0.21−2.92	0.72			
Statins	1.36	0.24−7.75	0.73			
LVEF	0.99	0.92−1.05	0.73			
Body fat percentage	1.02	0.94−1.11	0.60			
AC	0.99	0.79−1.24	0.92			
AMA	0.99	1.01−1.08	0.78			
CC	1.06	0.93−1.19	0.40			
Serum P	3.73	1.42−9.81	<0.01	3.46	1.17−10.24	0.02
Serum Ca	1.66	0.61−4.51	0.32			
Total cholesterol	1.00	0.98−1.02	0.78			
LDL-cholesterol	1.00	0.98−1.02	0.91			
Intact-PTH	1.00	0.99−1.01	0.50			
High sensitive CRP	1.89	0.77−4.63	0.17			
HbA1c	1.12	0.55−2.29	0.75			

AC, arm circumference; AMA, arm muscle area; BMI, body mass index; Ca, calcium; CC, calf circumference; CI, confidence interval; CRP, C-reactive protein; GNRI, geriatric nutritional risk index; HD, hemodialysis; LVEF, left ventricular ejection fracture; OR, odds ratio; P, phosphorus; LDL, low density lipoprotein; PTH, parathyroid hormone.

**Table 7:** Predictors of progression of lipid plaques.

Variables	Univariable analysis			Multivariable analysis		
	OR	95% CI	p value	OR	95% CI	p value
Malnutrition of GNRI	0.16	0.03−0.87	0.03	0.37	0.05−2.68	0.32
HD periods	0.99	0.99−1.00	0.22			
Age	1.06	0.96−1.17	0.23			
Male	0.88	0.07−10.7	0.92			
BMI	1.44	1.05−1.99	0.03	1.19	0.75−1.89	0.44
AC	1.51	1.03−2.20	0.03	1.19	0.67−2.12	0.54
AMA	1.10	0.99−1.22	0.09			
CC	1.01	0.91−1.12	0.83			
Body fat percentage	1.07	0.98−1.18	0.12			
Hypertension	1.14	0.19−6.89	0.89			
History of malignancy	-	-	1.00			
Current smoker	0.82	0.21−3.30	0.78			
Statins	0.87	0.14−5.54	0.88			
LVEF	1.04	0.97−1.12	0.3			
Total-cholesterol	1.00	0.99−1.03	0.53			
LDL cholesterol	1.00	0.98−1.02	0.74			
Triglyceride	1.00	0.99−1.01	0.81			
High sensitive CRP	0.77	0.35−1.69	0.51			
HbA1c	2.27	0.97−5.32	0.06			

AC, arm circumference; AMA, arm muscle area; BMI, body mass index; CC, calf circumference; CI, confidence intervals; CRP, C-reactive protein; GNRI, geriatric nutritional risk index; HD, hemodialysis; LDL, low-density lipoprotein; LVEF, left ventricular ejectin fraction; OR, odds ratio.

## Discussion

This study yielded important findings regarding the changes in coronary atherosclerotic plaque formation and non-culprit lesion morphology under different nutritional statuses and standard medical treatment in patients on maintenance HD. In culprit lesions that were amenable to PCI, the volume of the calcified plaques and calcified nodules were larger in the malnutrition state, and the plaque volume of lipid plaques was smaller. In addition, the lipid plaque progression was observed in the non-malnutrition group under anti-atherosclerotic treatment with agents such as statins. Conversely, a low nutritional status did not result in the progression of lipid plaques, but plaque progression was observed in calcified plaques. Multivariate regression analysis showed that low nutritional status and serum phosphorus levels were independent predictors of calcified plaque progression.

The high prevalence of CAD in HD patients is partly due to the concentration of risk factors for atherosclerosis [18]. Moreover, nutritional disorders, length of hemodialysis, oxidative stress, inflammation, and metabolic abnormalities such as homocysteinemia, hyperphosphatemia, and hypercalcemia are also considered non-traditional risk factors for atherosclerosis and calcification [14,19–22]. The longer a patient remains on dialysis, the more likely the problems of low nutrition and frailty emerge [16]. Decreased physical activity and nutritional impairment are additional determinants of cardiovascular events and prognosis [14,17]. However, no vivo studies using OCT have reported the effects of malnutrition and frailty on coronary artery atherosclerosis, and we believe that this study could demonstrate these effects.

First, OCT was used to evaluate significant stenotic lesions that were amenable to PCI, followed by grouping according to nutritional status. For significant stenotic lesions, the non-malnutrition group had larger lipid plaques, whereas the malnutrition group had larger calcified plaques and calcified nodules. Thus, malnutrition may affect the vascular calcification of the coronary arteries. However, we believe that this was a temporal evaluation and that it was necessary to observe plaque changes over time. Therefore, we decided to use OCT to evaluate the rate of plaque change over 6 months. The neointima of the drug-eluting stent implanted in culprit lesions did not differ significantly between the two groups, but the quantitative evaluation of non-culprit lesions showed a significant intergroup difference in the length of the lipid plaque and angle of the calcified plaque. Furthermore, the calcified plaque showed more advanced vascular calcification in a malnourished state.

### Vascular calcification

Here, comparisons between the two groups showed significant progression of vascular calcification in the malnutrition group, and multivariate analysis showed that "malnutrition" and "serum phosphorus level" were factors influencing the progression of vascular calcification. Calcification and necrotic components increase with the progression of chronic kidney disease [18]. Vascular calcification is an independent factor in CAD, and is also associated with the prognosis [23]. Thus, for HD patients, controlling vascular calcification is crucial for reducing CAD events and improving prognosis. In coronary arteries, there are two types of vascular calcification lesions: 1) atherosclerotic intimal calcification and 2) Menckeberg-type tunica media calcification. Patients undergoing HD and diabetic patients characteristically show Menckeberg-type tunica media calcification [24]. Although the mechanism underlying intimal calcification is not fully understood, it is currently thought to be an active process that begins within the lipid pool and involves the apoptosis of smooth muscle cells and macrophages and the release of matrix vesicles that calcify in the extracellular environment [25]. Mesangial calcification, on the other hand, is not influenced by intimal calcification and begins within the elastic fibers and smooth muscle cells of the tunica media, independent of lipid deposition and inflammation [25]. These smooth muscle cells lose their contractile properties, acquire osteochondral markers, and form bands of calcium-rich deposits that extend deep into the inner layer of the tunica media and may extend to the periphery of blood vessels [26]. In the advanced stages, calcification progresses to form solid plates or sheaths that increasingly distort the inner structure and invade the intima. The presence of bone proteins and cartilage in the vessel wall, as well as cells that exhibit osteoblastic differentiation, may indicate that vascular calcification shares similar processes with bone formation [27]. Oxidative stress, inflammation, tumor necrosis factor-α, hyperglycemia, peroxides, and hyperphosphatemia cause vascular calcification. Dialysis patients show abnormal phosphorus metabolism, resulting in hyperphosphatemia. Phosphorus has been widely reported to promote calcification of vascular smooth muscle [28]. Therefore, strict control of serum phosphorus levels is required to inhibit vascular calcification in HD patients. The relationship between hypotrophy and atherosclerosis/calcification has been reported in the malnutrition-inflammatory atherosclerosis syndrome, which is known as an inflammatory mediator mechanism [29]. Malnutrition suppresses vascular calcification inhibitors. This study demonstrated for the first time in vivo that malnutrition is associated with the progression of vascular calcification in coronary arteries. Thus, control of serum phosphorus levels and improvement of nutritional status are required to control calcification and improve the prognosis of HD patients.

### Lipid plaques

In this study, a 6-month longitudinal OCT evaluation showed that the length of the lipid plaques decreased in the malnutrition group and increased in the non-malnutrition group. In the univariate analysis, non-malnutrition, BMI, and arm circumference were significant risk factors, but none showed significance in the multivariate analysis. The significant factors in the univariate analysis alluded that weight gain and obesity were linked to the risk of lipid plaque progression. Although there are no clear criteria yet, it has been reported that an excessively high BMI is also associated with accelerated atherosclerosis and worsened mortality [30]. Prevention of malnutrition is important; however, proper weight and nutrition control is also required.

In vascular atherosclerosis, it is difficult to distinguish the development of lipid plaque from that of calcified plaque. This is due to the fact that calcification is considered to be the terminal phase of atherosclerosis. Especially in hemodialysis patients, there is a theory, known as the cholesterol paradox, which proposes that suppressing cholesterol with statins does not necessarily suppress arteriosclerosis [31]. Besides, in the field of basic research, it has been reported that low albumin or other low nutritional intake decreases vascular calcification inhibitory factors [32,33], and increased serum phosphorus levels induce the transformation of vascular smooth muscle cells, which in turn induce osteoblast differentiation [34]. It is also true that vascular calcification has a development mechanism other than normal atherosclerosis. Therefore, the results of the present study on the development of vascular calcification in low nutrition are consistent. Progressive calcification can cause poor stent dilatation and crimping during PCI and is a risk factor for increased revascularization and early and late stent thrombosis. Therefore, reducing the progression of calcification is clinically important as it improves PCI outcomes and prognosis [35,36].

This study demonstrates the importance of maintaining good nutritional status and the management of electrolytes such as phosphorus. We believe that the nutritional status of patients can be checked using nutritional screening and other methods, and that maintaining the nutritional status of patients can contribute to the prevention of atherosclerosis.

### Limitations

This study has few limitations. It was a small, single-center study and included only HD patients. We followed up only at 6 months and did not analyze for longer periods. In addition, the use of the plaque angle for quantitative assessment may have resulted in an underestimation of the amount of plaque. Furthermore, the OCT images did not include the entire length of the coronary artery but only evaluated the lesion area. Finally, because this study included patients with stable angina who required invasive coronary angiography, these data cannot be extrapolated to asymptomatic patients or patients presenting with acute coronary syndrome.

## Conclusion

In this study, we found that a low nutritional status likely affected the progression of coronary artery vascular calcification in maintenance HD patients. Appropriate management of nutritional status is crucial for suppressing the progression of CAD in HD patients. The findings in this study may be helpful for future HD management.

## Supporting information

S1 Raw data (XLSX)
